# Supplementary material for: The genome sequence of the fen violet, Viola stagnina Kit. ex Schult. (Malpighiales: Violaceae)
Source: Wellcome Open Res. 2026 Sep 28;11:771. [Version 1] doi: 10.12688/wellcomeopenres.27960.1 (PMC13740460; doi:10.12688/wellcomeopenres.27960.1)
Supplement: Peer review history

# Reviewer report: Reviewer response for version 1

**DOI:** 10.21956/wellcomeopenres.30760.r177860

**Published:** 2026-11-05

**Authors:** Aditya Nugroho

**Affiliations:** 1 Research Center for Applied Botany, National Research and Innovation Agency (BRIN), Cibinong Science Center Complex-BRIN, Cibinong, Bogor, Indonesia

The abstract provides a concise summary of the assembly size and chromosomal representation. However, including a key assembly quality metric, such as BUSCO completeness or consensus quality value, would help readers assess the reliability of the generated reference genome. 

 The background provides useful information on the distribution, conservation status, and biology of Viola stagnina. The authors could further strengthen the rationale by briefly explaining how a chromosome-level reference genome may facilitate future population genomic studies or conservation genetic research on this species.

Are the datasets clearly presented in a usable and accessible format, and the assembly and annotation available in an appropriate subject-specific repository?

Yes

Are sufficient details of the sequencing and extraction, software used, and materials provided to allow replication by others?

Yes

Are the rationale for sequencing the genome and the species significance clearly described?

Yes

Are the protocols appropriate and is the work technically sound?

Yes

Reviewer Expertise:

Plant genomics and molecular biology, with a particular focus on genome assembly, organellar genomics, next-generation sequencing, transcriptomics, and bioinformatics analysis of forest tree species.

I confirm that I have read this submission and believe that I have an appropriate level of expertise to confirm that it is of an acceptable scientific standard.

# Reviewer report: Reviewer response for version 1

**Published:** 2026-09-28

**Authors:** Automated Benchmarking

**Affiliations:** 1 Wellcome Sanger Institute, Hinxton, UK

As this article is a Genome Note, Automated Benchmarking has been used to determine whether the reported assembly passes four defined checks established by the Earth BioGenome Project: the raw and assembled data are publicly available, the Earth BioGenome Project three-part metric is met, the k-mer completeness exceeds 90%, and the BUSCO score passes the stated threshold. For Viola stagnina (ddVioStag1), all four checks passed. Data available: pass. ENA study PRJEB80116, raw-read accessions ERR13698300 and ERR13670061, and assembly accessions GCA_965234175.1 and GCA_965234045.1 are public. Earth BioGenome Project three-part metric: pass. The primary assembly is reported as 6.C.Q67, meeting the 6.C.Q40 reference standard. k-mer completeness: pass. The combined haplotype assemblies have 98.67% k-mer completeness, exceeding the Earth BioGenome Project target of 90%. BUSCO: pass. The primary assembly, which is proposed as the reference assembly for this species, has 98.4% complete BUSCOs (22.3% single-copy and 76.1% duplicated). BUSCO software version: 5.7.1; lineage dataset: eudicots_odb10. It meets the plant-specific target of greater than 90% complete BUSCOs. This decision uses values reported in the Genome Note and public accession records.

Reviewer Expertise:

NA

I confirm that I have read this submission and believe that I have an appropriate level of expertise to confirm that it is of an acceptable scientific standard.
